# Lipid changes in HIV-patients switching to the coformulated single tablet FTC/RPV/TDF (Eviplera^®^). Efficacy and safety analysis. GeSida Study 8114

**DOI:** 10.7448/IAS.17.4.19795

**Published:** 2014-11-02

**Authors:** Isabel A Pérez-Hernández, Rosario Palacios, Marisa Mayorga, Carmen M González-Doménech, Manuel Castaño, Antonio Rivero, Alfonso del Arco, Fernando Lozano, Jesús Santos

**Affiliations:** 1 Infectious Diseases, Hospital Virgen de la Victoria, Malaga, Spain; 2 Infectious Diseases, Hospital Carlos Haya, Malaga, Spain; 3 Infectious Diseases, Hospital Reina Sofía, Cordoba, Spain; 4 Internal Medicine, Hospital Costa del Sol, Marbella, Spain; 5 Infectious Diseases, Hospital Valme, Sevilla, Spain

## Abstract

**Introduction:**

Rilpivirine (RPV) has a better lipid profile than efavirenz (EFV) in naïve patients [1]. Switching to RPV may be convenient for many patients, while maintaining a good immunovirological control [2]. The aim of this study was to analyze lipid changes in HIV-patients at 24 weeks after switching to Eviplera^®^ (emtricitabine/RPV/tenofovir disoproxil fumarate [FTC/RPV/TDF]).

**Materials and Methods:**

Retrospective, multicentre study of a cohort of asymptomatic HIV-patients who switched from a regimen based on 2 nucleoside reverse transcriptase inhibitors (NRTI)+protease inhibitor (PI)/non nucleoside reverse transcriptase inhibitor (NNRTI) or ritonavir boosted PI monotherapy to Eviplera^®^ during February-December, 2013; all had undetectable HIV viral load for ≥3 months prior to switching. Patients with previous failures on antiretroviral therapy (ART) including TDF and/or FTC/3TC, with genotype tests showing resistance to components of Eviplera^®^, or who had changed the third drug of the ART during the study period were excluded. Changes in lipid profile and cardiovascular risk (CVR), and efficacy and safety at 24 weeks were analyzed.

**Results:**

Among 305 patients included in the study, 298 were analyzed (7 cases were excluded due to lack of data). Men 81.2%, mean age 44.5 years, 75.8% of HIV sexually transmitted. 233 (78.2%) patients switched from a regimen based on 2 NRTI+NNRTI (90.5% EFV/FTC/TDF). The most frequent reasons for switching were central nervous system (CNS) adverse events (31.0%), convenience (27.6%) and metabolic disorders (23.2%). At this time, 293 patients have reached 24 weeks: 281 (95.9%) have continued Eviplera^®^, 6 stopped it (3 adverse events, 2 virologic failures, 1 discontinuation) and 6 have been lost to follow up. Lipid profiles of 283 cases were available at 24 weeks and mean (mg/dL) baseline vs 24 weeks are: total cholesterol (193 vs 169; p=0.0001), HDL-c (49 vs 45; p=0.0001), LDL-c (114 vs 103; p=0.001), tryglycerides (158 vs 115; p=0.0001), total cholesterol to HDL-c ratio (4.2 vs 4.1; p=0.3). CVR decreased (8.7 vs 7.5%; p= 0.0001). CD4 counts were similar to baseline (653 vs 674 cells/µL; p=0.08), and 274 (96.8%) patients maintained viral suppression.

**Conclusions:**

At 24 weeks after switching to Eviplera^®^, lipid profile and CVR improved while maintaining a good immunovirological control. Most subjects switched to Eviplera^®^ from a regimen based on NNRTI, mainly EFV/FTC/TDF. CNS adverse events, convenience and metabolic disorders were the most frequent reasons for switching.
